# Leucocyte- and Platelet-Rich Fibrin Block: Its Use for the Treatment of a Large Cyst with Implant-Based Rehabilitation

**DOI:** 10.3390/medicina57020180

**Published:** 2021-02-20

**Authors:** Rodolfo Mauceri, Denise Murgia, Orazio Cicero, Luigi Paternò, Luca Fiorillo, Viviana De Caro, Giuseppina Campisi

**Affiliations:** 1 Department of Surgical, Oncological and Oral Sciences, University of Palermo, 90127 Palermo, Italy; denise.murgia@unipa.it (D.M.); campisi@odonto.unipa.it (G.C.); 2 Department of Biomedical and Dental Sciences, Morphological and Functional Images, University of Messina, Policlinico G. Martino, 98100 Messina, Italy; lucafiorillo@live.it; 3 Department of Biological, Chemical and Pharmaceutical Sciences and Technologies, University of Palermo, 90123 Palermo, Italy; viviana.decaro@unipa.it; 4 Private Practice, 96019 Rosolini, Italy; oraziocicero@libero.it (O.C.); ambulatorio_paterno@virgilio.it (L.P.)

**Keywords:** platelet-rich fibrin, PRF, PRF block, bone regeneration, odontogenic cyst, case report

## Abstract

The management of critical-size bone defects is still demanding. Recently, autologous platelet concentrates in combination with bone substitute have been applied and reported in a few studies. Our aim is to report the healing of a critical-size alveolar bone defect treated with a new bone regeneration technique by means of L-PRF and L-PRF blocks. A 45-year-old woman presented a large cystic lesion; the extraction of three teeth, a cyst removal procedure, and bone regeneration procedures with L-PRF and L-PRF blocks were planned. The L-PRF block was prepared by mixing a bone substitute with a piece of L-PRF membrane and liquid fibrinogen. Additionally, after bone healing an implant-based rehabilitation was optimally performed. On the basis of the positive results, in terms of bone healing and tissue regeneration in a large bone defect, the application of L-PRF and L-PRF blocks, in agreement with the scarce literature, is suggested as a feasible procedure in selected cases.

## 1. Introduction

Tooth loss is known to be related to a bone resorption process that can significantly affect alveolar bone volume. Moreover, if in the presence of a large alveolar bone cyst, the cyst enucleation combined with treatment tooth extraction may end with critical-size defects whose healing may be incomplete due to the size of the defect and the loss of periosteum or bone walls [1,2].

To prevent the development of severe bone resorption and provide an adequate bone height and width for successive implant rehabilitation, numerous surgical techniques have been proposed, such as alveolar ridge preservation (ARP) or guided bone regeneration procedures (GBR) [3].

Recently, autologous platelet concentrates (APC) have been applied to provide more predictable results in several oral surgical procedures due to their ability to enhance soft and hard tissue regeneration, positively influencing wound healing [4,5,6,7].

Leukocyte- and platelet-rich fibrin (L-PRF), a strong fibrin matrix produced by the centrifugation of autologous blood, contains cytokines and growth factors such as platelet-derived growth factor (PDGF), vascular endothelial growth factor (VEGF), and transforming growth factor beta (TGF-b), and shows several healing properties such as angiogenesis, wound protection, and immune control [5,6]. 

Recently, the use of a fluid form of PRF has been proposed in order to bond particulate bone substitute, imbibe it with PRF growth factors, and make it more adhesive and stable [8]. The result of this procedure is called L-PRF block [9]. 

The aim of this report was to evaluate the healing of a critical-size alveolar bone defect treated with a new bone regeneration technique by means of L-PRF, as a membrane, and L-PRF block, in order to maintain the bone volume after the extraction of three dental elements and the removal of a large cystic lesion.

## 2. Case Report

A non-smoker 45-year-old woman complained of right-side facial pain and swelling, which had begun some days before the first clinical examination.

The clinical examination revealed abnormal mobility of teeth #14–16 and pain response to percussion testing. There was no significant systemic complaint. The family history was unremarkable.

**Figure 1:**
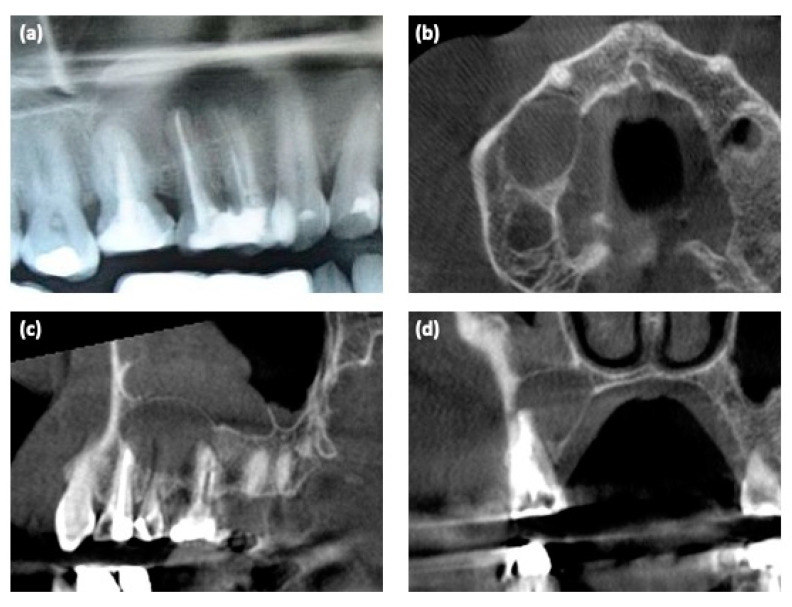
Radiological examinations. (**a**) Detail of the dental X-ray at the first clinical examination; (**b**–**d**) 3D CBCT scan before surgery.

The dental X-ray showed the presence of a large cystic lesion related to teeth #14–16, which previously had been endodontically treated (Figure 1a). Thus, to evaluate the 3D dimensions of the lesion, cone-beam computed tomography (CBCT) with a multiplanar reconstruction program was used. The CBCT showed a periapical lesion of approximately 20 × 15 × 14 mm, with a soft, fluctuant, and cystic consistency, resulting in a critical-size alveolar bone defect [1,10] (Figure 1b–d). 

The final diagnosis was odontogenic cyst and teeth #14–16 with a hopeless prognosis.

Based on the findings, teeth extraction, cyst removal, and GBR procedures combined with L-PRF and L-PRF block were planned.

Before the beginning of the surgical procedures, L-PRF and PRF block were prepared according to the techniques described by Dohan et al. (2009) [11] and Cortellini et al. (2018) [9], respectively. Venous blood was collected from the patient in 8 tubes of 10 mL. Two tubes with a white cap and glass coating (WCT; IntraSpin™, Intra-Lock, FL, USA) and six tubes with a red cap and plastic coating (BVBCTP2; IntraSpin™, Intra-Lock, FL, USA) were used and centrifugated in the IntraSpin™ system (ISS220 Intra-Lock, FL, USA) at 2700 rpm. The white cap tubes required 3 min of centrifugation in these conditions to produce the Liquid Fibrinogen (PLyF) at the top, which was aspirated with a sterile syringe without the red part; to obtain L-PRF in the red cap tubes, a further 9 min (for a total of 12 min) was required.

In the red cap tubes, three layers were formed after centrifugation: a base of red blood cells at the bottom; acellular plasma on the top (supernatant); and, finally, a clot of PRF between them. The L-PRF clots were then withdrawn by sterile tweezers and isolated from the red blood cells (RBCs), which, as platelet-poor plasma (PPP), were discarded. To obtain L-PRF membrane, the clots were moved on the grill of the Xpression Box (IntraSpin™, Intra-Lock, FL, USA) and pressed for about 5 min. The exudate was collected and kept for next steps. 

The PRF-block was then prepared. Two L-PRF membranes were cut into small pieces and mixed to 0.5 g of OSP-OX31 osteOXenon Mix Granules (Bioteck S.p.A, Arcugnano Italy) (50:50 ratio); the PLyF and compression exudate were then added to the system, stirred gently for about 10 s, and shaped to obtain a homogeneous complex in the desired form. The activated platelets of the chopped membranes let fibrinogen polymerize into fibrin within few minutes, entrapping the biomaterial into a fibrin network containing platelets and leucocytes.

**Figure 2:**
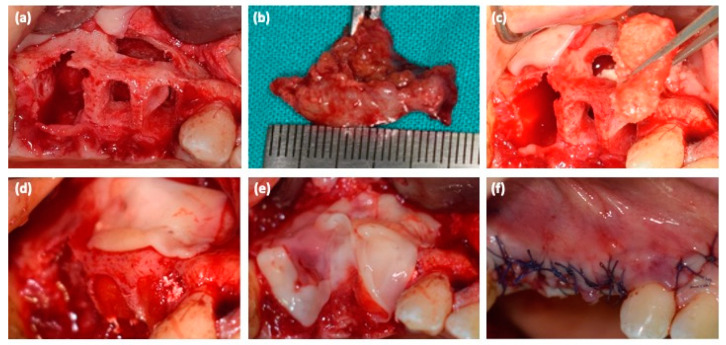
(**a**) bone defect after dental extractions and cyst removal; (**b**) cystic lesion; (**c**) the application of L-PRF clot under the sinus floor and L-PRF block in the bone lesion and post-extraction sockets; (**d**,**e**) the application of the remaining L-PRF clots over the surgical site; (**f**) sutures.

The surgical protocol included (1) chlorhexidine 0.2% mouthwash 30 mL swished up to 60 s; (2) local anesthesia, achieved using 3% mepivacaine hydrochloride without adrenaline; (3) the elevation of a full-thickness mucoperiosteal flap; (4) tooth luxation and avulsion, gently performed with elevators and forceps; (5) the removal of the cyst and debridement of the post-extraction socket with miller surgical curette and irrigation (Figure 2a,b); (6) the application of L-PRF clot under the sinus floor and L-PRF block in the bone lesion and post-extraction sockets (Figure 2c); (7) the application of the remaining L-PRF clots over the surgical site (Figure 2d,e); (8) tension-free soft tissue closure (Figure 2f). Sutures were removed after 10 days. The post-operative protocol provides analgesics therapy (i.e., paracetamol 500 mg tablet thrice a day, if needed) and chlorhexidine 0.2% mouthwash (thrice a day for 10 days).

## 3. Results

Histological examination confirmed the odontogenic nature of the cyst (i.e., periapical/radicular cyst). In the following weeks, the patient was under strict oral hygiene measures and on regular recall. Tissue healing was uneventfully; there was no infection or swelling after the surgery and patient did not complain particularly about pain or discomfort.

After 10 days, once the sutures were removed, the L-PRF membranes were partially reabsorbed.

**Figure 3:**
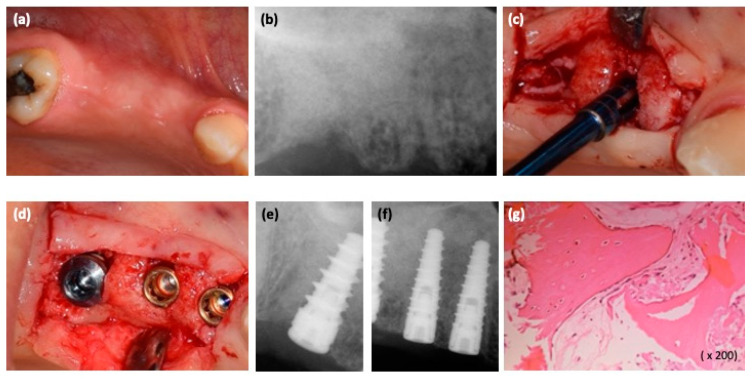
(**a**,**b**) Six months post-surgical regeneration procedures, clinical and radiological view; (**c**) bone sample harvesting procedure at the #15 implant site using a trephine bur; (**d**) dental implant placement; (**e**,**f**) intraoral radiograph after placing dental implants; (**g**) the magnification of histological findings of the bone sample (osteoblastic cells on the surface of the new woven bone containing osteocytes).

Six months postoperatively, the bone tissue healing was observed with dental X-ray (Figure 3a,b).

The second stage surgery was then performed; before implant placement, using a trephine bur, an alveolar bone sample was obtained from the #15 implant site (Figure 3c). Three implants (Blossom, Intra-lock, Birmingham, AL, USA) were placed in the area of teeth #14–16 (Figure 3d–f). The sample was processed and bone histomorphometry was performed (Figure 3g). Sutures were removed after 10 days.

**Figure 4:**
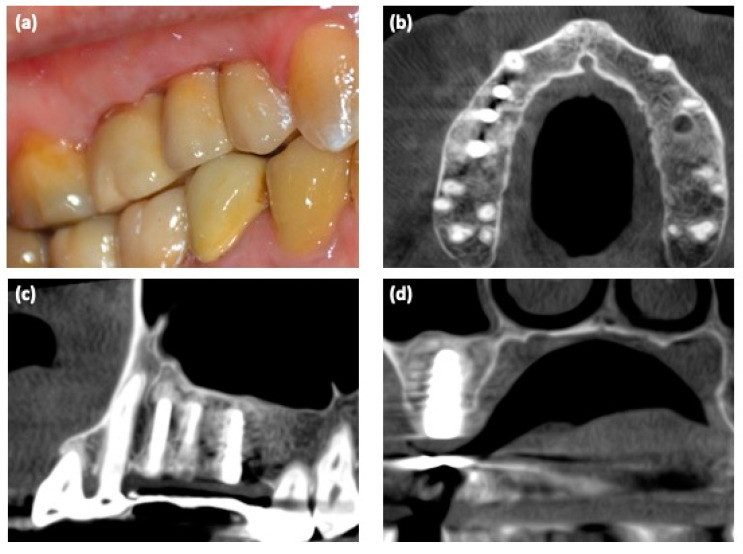
(**a**) Clinical view of the implant-based rehabilitation; (**b**–**d**) 3D CBCT scan after implant placements.

Three months after the implant placement, an impression was made; implants were loaded with screw-retained temporary fixed partial restorations. Definitive restoration was placed after 2 months (Figure 4a). The regeneration of bone tissue was highlighted with a new CBCT (Figure 4b–d).

## 4. Discussion

The purpose of this report was to describe the healing of a wide alveolar defect with the application of L-PRF in its pure form and as an L-PRF block. 

Different GBR and ARP techniques have been proposed to prevent bone resorption. Among the graft materials currently used in oral procedures, autologous bone blocks are described as the gold standard. However, the need for a second oral surgical site, especially if outside the oral cavity, is related to a higher patient morbidity and discomfort.

The application of several bone substitutes in dentistry has been proposed, with heterogeneous results. The recent findings rely on the advantages of APC to enhance the properties of the bone substitute [5,6].

L-PRF is a second-generation platelet concentrate; its strong architecture composed of micropores and thin fibers serves as a scaffold for osteogenesis cell migration, differentiation, and neovascularization, maintaining the slow release of growth factors up to a period of 7–14 days [11,12]. Moreover, L-PRF reduces post-operative pain and swelling and acts as a barrier membrane, reducing the cost of surgical procedures [12]. For these interesting properties, it has been used also for the management of several oral surgical problems, such as facial cutaneous sinus tracts secondary to medication-related osteonecrosis of the jaw [13].

To enhance the new bone formation, biocompatible bone graft material was processed with L-PRF membranes and fixed with liquid fibrinogen to obtain the L-PRF block. The L-PRF acted in the L-PRF block as a biological connector among the graft particles, creating a matrix with suitable manipulative qualities that was rich in bioactive molecules and growth factors [8,9,11,14].

Few authors have reported cases of PRF block in human oral surgeries; only 26 interventions in 15 cases have been reported [9,15,16]. These cases are reported in Table 1. 

Chenchev et al. evaluated the possibility of the augmentation of the alveolar ridge in the frontal region of the upper jaw in a 18-year old male. The authors applied a combination of bone graft materials, injectable platelet-rich-fibrin (i-PRF), and advanced platelet-rich fibrin (A-PRF). The augmentation procedure was successful and the dental implant was placed [14].

In a single-cohort observational study, Cortellini et al. performed 15 bone augmentation procedures on 10 patients. All the patients (mean age 50.7 years) were affected by horizontal bone defect; the surgical procedures provide the application of L-PRF block (a combination of two L-PRF membranes, 0.5 deproteinized bovine bone mineral, and liquid fibrinogen), collagen membrane, and L-PRF membranes. Significant horizontal ridge augmentation was obtained with the L-PRF block; which allowed implant placement in all cases [9].

Andrade et al. published a pilot clinical study on four patients; they applied a “dentin block” (a mixture of particulate autologous dentin with chopped L-PRF membranes and liquid fibrinogen) in ten extraction sockets. The “dentin block” was able to promote new bone formation, without host tissue reactions, and a favorable dentin resorption/bone formation rate. All the patients demonstrated an adequate amount and quality of bone for implant placement [15].

To our knowledge, our case report is the first case regarding the application of L-PRF and L-PRF block for the management of large cystic removal and extraction of three teeth.

The abovementioned beneficial properties of L-PRF played a key role in remineralization, which was already significant six months after the application.

Even if there is no one standard definition of critical-sized defects, alveolar bone defects measuring 2–3 cm in diameter usually heal in about 12 months [1,2].

In our case, the bone healing was achieved in about six months; moreover, our histological analysis showed that the L-PRF block was able to produce mature alveolar bone tissue. Indeed, the regenerated bone structure allowed the subsequent implant prosthetic rehabilitation.

Despite all the limitations of reporting a case, the positive outcomes in terms of bone healing and tissue regeneration reinforced the effective role of L-PRF in oral surgical procedures.

## 5. Conclusions

The present case report shows that the addition of L-PRF and L-PRF blocks may be successful for bone regeneration procedures also in critical-size defects, supporting the data found in the literature regarding the advantages of APC and especially of L-PRF. Still, further research and randomized controlled trials are necessary to confirm the results presented.

## Figures and Tables

**Table 1:** Reported cases of PRF block used in human oral bone regeneration procedures.

Authors (Years)	N. of Patients	Age (yr)	Sex	Type of Intervention	Type of APC	Type of Bone Substitute for PRF Block	N. of Intervention	Outcome
Chencev IL et al. (2017) [15]	1	18	Male	Alveolar ridge augmentation	i-PRF	Bone graft material (nd)	1	It revealed successful augmentation of the alveolar ridge. The dental implant was placed in the area of tooth 11
Cortellini S et al. (2018) [9]	10	50.7	Nd	Bone augmentation procedures	L-PRF	Deproteinized bovine bone mineral	15	“Significant horizontal ridge augmentation was obtained with L-PRF Block. The resorption rate of the graft was very low, which allowed implant placement in all cases.”
Andrade C et al. (2020) [16]	4	54	Female	Alveolar ridge preservation	L-PRF	Dentin particles	10	“…dentin block was able to promote new bone formation, without host tissue reactions, and a favorable dentin resorption/bone formation rate. All patients demonstrated an adequate amount and quality of bone for implant placement.”
